# Clinical Outcomes of Penetrating Keratoplasty Performed with the VisuMax Femtosecond Laser System and Comparison with Conventional Penetrating Keratoplasty

**DOI:** 10.1371/journal.pone.0105464

**Published:** 2014-08-15

**Authors:** Kazutaka Kamiya, Hidenaga Kobashi, Kimiya Shimizu, Akihito Igarashi

**Affiliations:** Department of Ophthalmology, University of Kitasato School of Medicine, Kanagawa, Japan; Cardiff University, United Kingdom

## Abstract

**Purpose:**

To assess the clinical outcomes of femtosecond laser-assisted keratoplasty (FLAK) using the VisuMax femtosecond laser system, and to compare them with those of conventional penetrating keratoplasty (PK).

**Methods:**

We retrospectively examined 20 eyes of 20 consecutive patients undergoing FLAK and 20 eyes of 20 age- and diagnosis-matched patients undergoing conventional PK. We quantitatively assessed corneal astigmatism, refractive astigmatism, and corrected visual acuity, 1, 3, and 6 months postoperatively, and endothelial cell density 6 months postoperatively.

**Results:**

Corneal and refractive astigmatism after FLAK were significantly lower after FLAK than that after conventional PK at 3 and 6 months postoperatively (p = 0.04 and p = 0.03, respectively, Mann-Whitney U test). FLAK provided significantly faster visual recovery than conventional PK at 1 month postoperatively (p = 0.02), but not at 3 and 6 months postoperatively (p = 0.52 and p = 0.80, respectively). We found no significant differences in the change in endothelial cell density between the two groups (p = 0.30).

**Conclusions:**

FLAK using the VisuMax femtosecond laser system induces significantly less corneal and refractive astigmatism than conventional PK, and provides significantly faster visual recovery in the early postoperative period, possibly because the geometry of the donor-recipient matching is more physiological and requires less tight sutures. It is suggested that FLAK has advantages over conventional PK, in terms of astigmatism and fast visual recovery.

## Introduction

The femtosecond laser is one of the most revolutionary inventions in recent medical technology. In ophthalmology, it has been used mainly for the creation of corneal flaps for laser in situ keratomileusis as an alternative to the mechanical microkeratome. A recent breakthrough in this technology has enabled us to apply for corneal transplantation. With regard to penetrating keratoplasty (PK), there have been several studies on femtosecond laser assisted penetrating keratoplasty (FLAK). [1]–[18] However, there have so far been only a few published studies on FLAK using a straight cut with angles of 90° in a clinical setting, [3], [4] because the profiled trephinations for FLAK have been mainly focused on the mushroom, the top hat, and the zigzag profiles using another femtosecond laser system. [5]–[18] To our knowledge, no previous study on FLAK using VisuMax femtosecond laser system (Carl Zeiss Meditec, Jena, Germany) with a 500-kHz repetition rate has been reported. In addition, comparison of clinical outcomes of FLAK with a straight cut incision and conventional PK with manual trephination has not so far been conducted. The purpose of the present study is twofold; to retrospectively assess the clinical outcomes of FLAK using a 500-kHz VisuMax femtosecond laser system, and to compare them with those of conventional PK in terms of astigmatism, visual acuity, and endothelial cell density.

## Materials and Methods

Twenty eyes of 20 consecutive patients (12 men and 8 women) who underwent FLAK using the VisuMax femtosecond laser system with a 500 kHz repetition rate, and 20 eyes of 20 age- and diagnosis-matched patients (10 men and 10 women) who underwent conventional PK using manual trephination by the same surgeon, were included in this observational study. The subjects who underwent additional procedure such as cataract surgery were excluded from this study. The sample size in this study offered 87% statistical power at the 5% level in order to detect a 2.0-diopter (D) difference in corneal astigmatism, when the standard deviation (SD) of the mean difference was 2.0 D, and 80% statistical power at the 5% level in order to detect a 0.10-difference in the logarithm of the minimal angle of resolution (logMAR) of visual acuity, when the SD of the mean difference was 0.11. The patient ages at the time of surgery were 63.7±23.2 years (mean age ± SD; range, 17 to 88 years) in the FLAK group, and 64.3±14.8 years (range, 38 to 85 years) in the conventional PK group. Written informed consent was obtained from all patients for the surgery. This retrospective review of the data was approved by the Institutional Review Board at Kitasato University and followed the tenets of the Declaration of Helsinki. Our Institutional Review Board waived the requirement for informed consent for this retrospective study.

### Surgical procedures

For FLAK, we used the VisuMax femtosecond laser system (Carl Zeiss Meditec) with a 500 kHz repetition rate. The donor cornea was mounted on an artificial anterior chamber, and brought to the femtosecond laser, which was programmed to perform a straight cut with angles of 90° with certain depth and diameter based on the recipient cornea measurements. The femtosecond laser parameters were as follows: donor graft size 6.9 to 7.7 mm, recipient graft size 6.7 to 7.5 mm, 300 nJ power, with side cut angles at 90°, and spot size 3 µm. The donor cornea was oversized in diameter by 0.2 mm in all cases, according to the manufacturer’s instructions. An uncut gap of 25 µm was left in order to avoid corneal perforation during the laser cut. This gap was bluntly dissected during surgery using a Sinskey hook, and excision of the recipient corneal button was completed with Katzin curved corneal scissors.

For conventional PK, we used the standardized manual trephination technique. In brief, the donor button was punched from the endothelial side on curved blocks using the Hessburg-Barron vacuum trephine (Barron Precision Instruments, Grand Blanc, MI, USA). A Barron suction trephine was used to cut a partial depth, circular incision in the cornea, centered at the geometric center of the cornea. The donor cornea was oversized by 0.50 mm in all cases. Excision of the recipient corneal button was completed with curved corneal scissors.

In both groups, either 16 interrupted sutures (10-0 nylon), or 8 interrupted sutures and a single continuous 16-bite suture (10-0 nylon) were placed. All penetrating keratoplasties were performed under general anesthesia by the same surgeon (KK). After surgery, steroidal (0.1% betamethasone, Rinderon ™, Shionogi, Osaka, Japan) and antibiotic (0.5% levofloxacin, Cravit™, Santen, Osaka, Japan) medications were topically administered 4 times daily for 1 month, and then the frequency was steadily reduced. In both groups, the same postoperative treatment regimen was used. As our study protocol, suture removal was not generally done during the 6-month observation period, but loose sutures were removed upon diagnosis in all subjects.

### Assessment of Astigmatism, Visual Acuity, and Endothelial Cell Density

We assessed corneal astigmatism, refractive astigmatism, and logMAR (logarithm of the minimum angle of resolution) best spectacle-corrected visual acuity (BSCVA), 1, 3, and 6 months postoperatively, and corneal cell density 6 months postoperatively in each group, For the assessment of corneal astigmatism, the simulated keratometric readings were measured with corneal topography (ATRAS995™, Carl Zeiss Meditec). The endothelial cell density was determined with a non-contact specular microscope (SP-8800, Konan, Nishinomiya, Japan). The manufacturer's software automatically produced an endothelial cell density measurement by visually comparing the cell size in the image with the predefined patterns on the screen. We performed at least 3 measurements in each device, and the averaged values were used for statistical analysis. All examinations were performed by experienced ophthalmic technicians.

### Statistical Analysis

All statistical analyses were performed using StatView software version 5.0 (SAS, Cary, USA). The Mann-Whitney U test was used to compare the data between the two groups. The chi-square test was used to compare the primary diseases between the two groups. The results are expressed as mean ± SD, and a value of p<0.05 was considered statistically significant.

## Results

### Study Population

**Figure 1:**
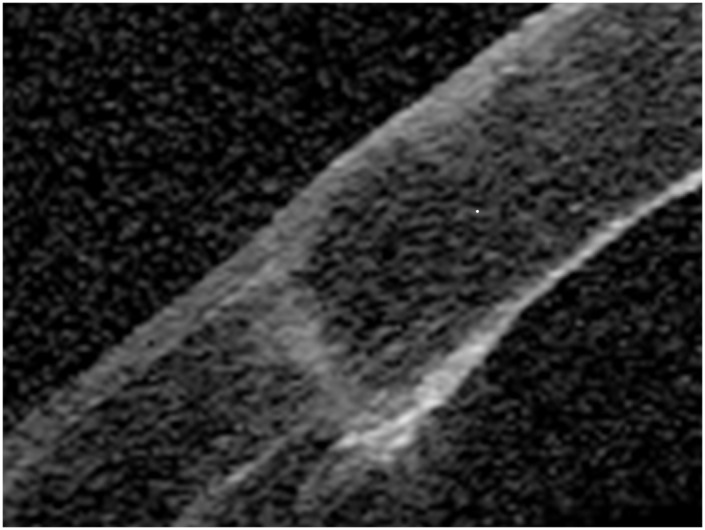
Anterior segment optical coherence tomography image after femtosecond laser assisted penetrating keratoplasty using a straight cut with angles of 90°.

Preoperative patient demographics and diagnosis are summarized in Table 1. There were no significant differences between the two groups in terms of age (p = 0.54, Mann-Whitney U test), gender (p = 0.53), logMAR UCVA (p = 0.97), or logMAR BSCVA (p = 0.58). The preoperative endothelial cell density obtained from the records of the eye banks that supplied the donor corneas was 2583±253 cells/mm^2^ and 2529±326 cells/mm^2^, in the FLAK and conventional PK groups, respectively (p = 0.23). The donor and recipient graft sizes were 7.1±0.2 and 6.9±0.2 mm, respectively, in the FLAK group. The corresponding figures were 7.5±0.1 and 7.0±0.1 mm, in the conventional PK group. No eyes were lost during the 6-month follow-up in this series. All surgical procedures were uneventful. In the FLAK group, no complications occurred in any eyes during laser trephination, during transportation of the patient from the laser suite to the operating room, or during the transplantation procedure in the operating room. Figure 1 shows a representative example of anterior segment optical coherence tomography image after FLAK.

**Table 1:** Preoperative demographics and diagnosis of the study population.

	FLAK group	Conventional PK group	P value
No. of patients	20	20	-
No. of eyes	20	20	-
Age	63.7±23.2 years (range, 17 to 88 years)	64.3±14.8 years (range, 38 to 85 years)	0.54
Gender (% female)	40.0	50.0	0.53
Preoperative UCVA (logMAR)	1.73±0.48 (range, 1.00 to 3.00)	1.77±0.67 (range, 0.52 to 3.00)	0.97
Preoperative BSCVA (logMAR)	1.45±0.70 (range, 0.40 to 3.00)	1.65±0.77 (range, 0.40 to 3.00)	0.58
**Primary disease**			
Keratoconus	6 (30%)	4 (20%)	0.93
Bullous keratopathy	5 (25%)	5 (25%)	
Prior ulcer/scarring	4 (20%)	6 (30%)	
Corneal dystrophy	2 (10%)	3 (15%)	
Fuchs’ dystrophy	1 (5%)	1 (5%)	
Herpetic disease	2 (10%)	1 (5%)	

FLAK = femtosecond laser assisted penetrating keratoplasty, PK = penetrating keratoplasty, UCVA = uncorrected visual acuity, logMAR = logarithm of the minimal angle of resolution, BSCVA = best spectacle-corrected visual acuity.

### Corneal and Refractive Astigmatism

**Figure 2:**
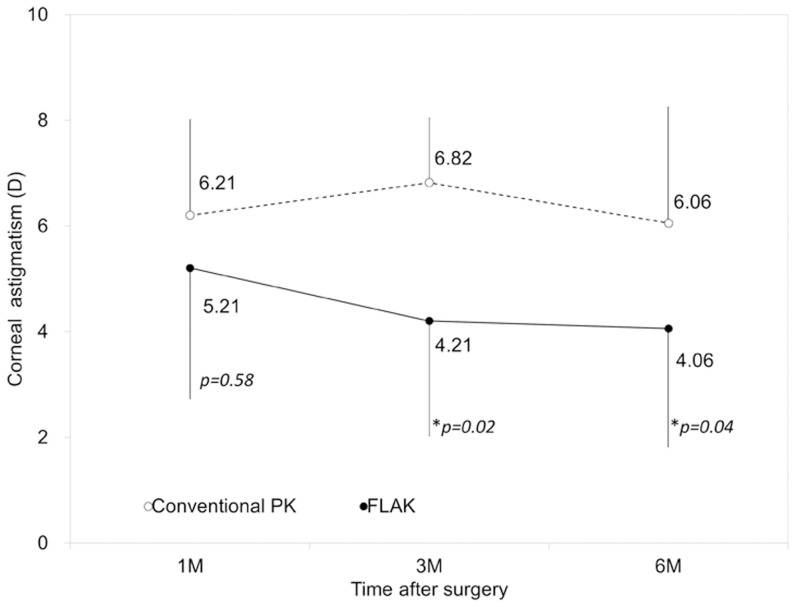
Time course of corneal astigmatism after femtosecond laser assisted keratoplasty (FLAK) and conventional penetrating keratoplasty (PK). *; p<0.05.

**Figure 3:**
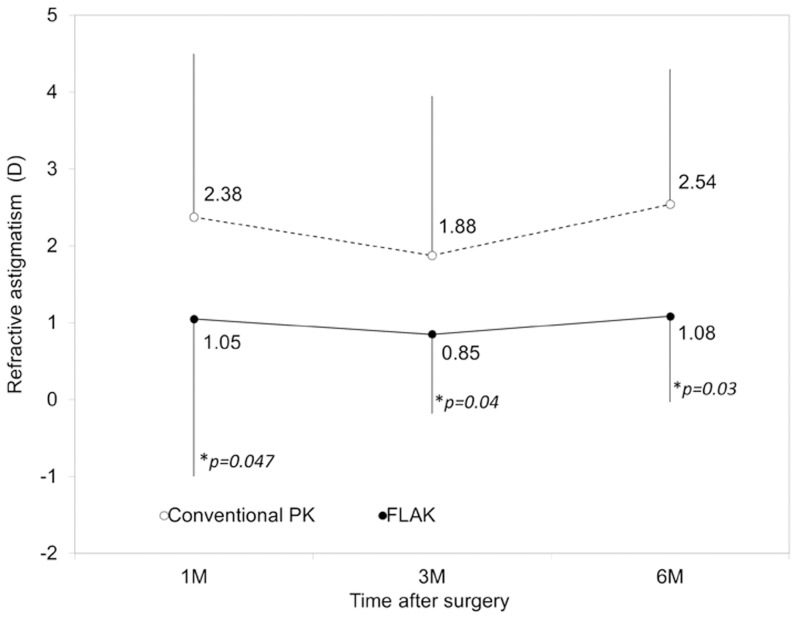
Time course of refractive astigmatism after femtosecond laser assisted keratoplasty (FLAK) and conventional penetrating keratoplasty (PK). *; p<0.05.

Time courses of corneal and refractive astigmatism are shown in Figures 2 and 3, respectively. Corneal astigmatism was significantly lower in the FLAK group than that in the conventional PK group at 3 and 6 months postoperatively (p = 0.02, and p = 0.04, respectively, Mann-Whitney U test), but not at 1 month postoperatively (p = 0.58). Refractive astigmatism in the FLAK group was also significantly lower in the FLAK group than that in the conventional PK group at 1, 3, and 6 months postoperatively (p = 0.047, p = 0.04, and p = 0.03, respectively, Mann-Whitney U test).

### Visual Acuity

**Figure 4:**
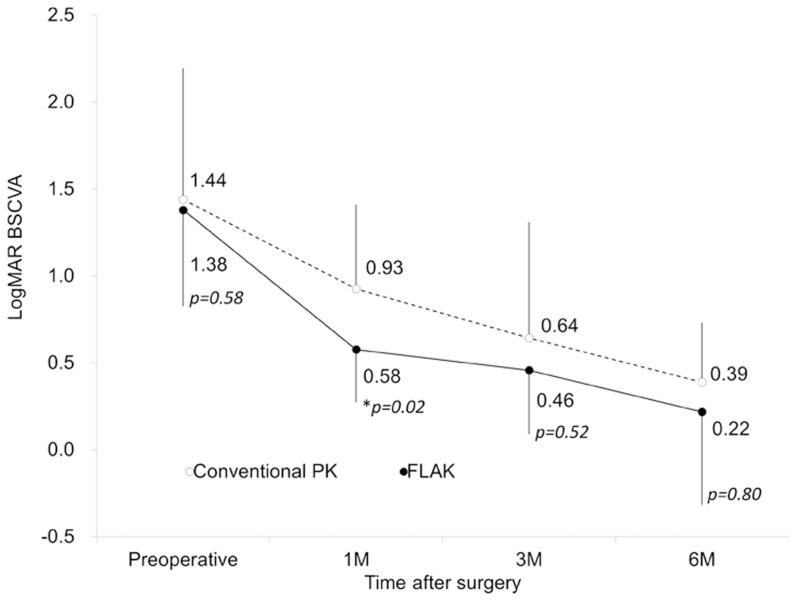
Time course of logarithm of the minimal angle of resolution (logMAR) best spectacle-corrected visual acuity (BSCVA) before and after femtosecond laser assisted keratoplasty (FLAK) and conventional penetrating keratoplasty (PK). *; p<0.05.

A subgroup of eyes with normal visual potential (with normal macular and optic nerve function) were included in the analysis of BSCVA. Time course of logMAR BSCVA are shown in Figure 4. The FLAK group showed significantly faster visual recovery than conventional PK group at 1 month postoperatively (p = 0.02), but there were no significant differences in logMAR BSCVA between the two groups at 3 and 6 months postoperatively (p = 0.52 and p = 0.80, respectively).

### Endothelial Cell Density

The endothelial cell density was 1892±455 cells/mm^2^ and 1869±644 cells/mm^2^, 6 months postoperatively, in the FLAK and conventional PK groups, respectively (p = 0.57). We found no significant difference in the change in endothelial cell density from preoperatively to 6 months postoperatively between the two groups (p = 0.30).

### Secondary Surgeries/Adverse Events

In the FLAK group, graft rejection developed in 1 eye (5%). In the conventional PK group, graft rejection, increased intraocular pressure, and suture-oriented infection developed in 2 eyes (10%), 3 eyes (15%), and 1 eye (5%), respectively. All these eyes were followed with appropriate medical therapy, and resolved thereafter. No other vision-threatening complications were seen at any time during the 6-month observation period.

## Discussion

In the present study, our results demonstrated that FLAK induces significantly less corneal and refractive astigmatism than conventional PK 6 months postoperatively, despite the fact that no suture removal was routinely performed. Although we present a comparatively small amount of sample data, and the follow-up time is short, our results are comparable with previous FLAK studies, as shown in Table 2, [4], [5], [7]–[9], [11]–[13], [16]–[18] suggesting that FLAK using the VisuMax femtosecond laser system provides almost equivalent clinical outcomes to that using other femtosecond laser system. As far as we can ascertain, this is the first study to compare the clinical outcomes of FLAK using a straight cut with angles of 90° with those of conventional PK. There have been only a few published studies on comparison of the clinical outcomes of FLAK and conventional PK, and all FLAK procedures were performed using the Intralase femtosecond laser system (Abbott Medical Optics, Abbot Park, IL, USA) with a zigzag cut configuration. [13], [16], [17] Farid et al demonstrated that FLAK had significant improvement in astigmatism compared with conventional PK at 1 and 3 months postoperatively, but not after the 6-month postoperative follow-up period. [13] Chamberlain et al reported that significantly lower topographic astigmatism was achieved in the FLAK group over the conventional PK group in the 4- to 6-month follow-up period, but that this difference in astigmatism was no longer present at any other follow-up period up to 24 months postoperatively. [16] Gaster et al showed that topographic astigmatism 6 months postoperatively was 4.76±3.41 D and 5.91±4.53 D, in the FLAK and conventional PK groups, respectively. [17] Our findings were in accordance with these previous studies demonstrating that FLAK induces less corneal astigmatism than conventional PK.

**Table 2:** Clinical outcomes of femtosecond laser assisted penetrating keratoplasty (FLAK).

	Number ofeyes	Femtosecondlaser	Incision	Age	Follow-up(months)	BSCVA	Cornealastigmatism (D)	Refractiveastigmatism (D)	Endothelial cell density(cells/mm^2^)
Hoffart et al^4^	9	40-kHz Femtec	straight cut	68.9±16.5	6±3	0.54±0.22 logMAR	4.6±2.3	2.9±1.2	1194±465
Buratto et al^5^	7	15-kHz Intralase	top hat cut	46.9±13.8	3	20/60 to 20/20	NA	2.9±1.1	NA
Farid et al^7^	13	Intralase	zigzag cut	63.2±15.9	3 to 9	CF to 20/100	2.2±1.1	NA	NA
Price et al^8^	6	15-kHz Intralase	top hat cut	76.7±14.0	12	CF to 20/50	NA	5.4±2.5	2030±600
Por et al^9^	8	10-kHz Femtec	straight cut	60.3	9.5	CF to 20/20	NA	2.56	NA
Cheng et al^11^	5	Intralase	top hat cut	42 to 75	12	20/32	3.26±2.1	3.20±2.0	1793±491
Bahar et al^12^	16	60-kHz Intralase	top hat cut	42.2±18.7	9.9±3.7	0.32±0.31 logMAR	NA	3.6±1.9	1981±474
Farid et al^13^	49	60-kHz Intralase	zigzag cut	59.82	1 to 12	0.2 logMAR	2.9±1.0	NA	NA
Chamberlain et al^16^	50	60-kHz Intralase	zigzag cut	46.26	11.85	0.265 logMAR	6.06	NA	NA
Gaster et al^17^	54	60-kHz Intralase	zigzag cut	38.7	6	0.44±0.49 logMAR	4.76±3.71	NA	NA
Birnbaum et al^18^	32	60-kHz Intralase	mushroom cut	39±11	14.1±5.1	0.26±0.41 logMAR	6.2±3.6	5.3±3.8	19.0% loss/year
	91	60-kHz Intralase	top hat cut	57±11	10.7±5.1	0.34±0.28 logMAR	6.0±4.0	5.1±2.5	16.5% loss/year
Current	20	500-kHz VisuMax	straight cut	63.7±23.2	6	0.22±0.53 logMAR	4.06±2.24	1.08±1.11	1775±422

BSCVA = best-spectacle corrected visual acuity, D = diopter, logMAR = logarithm of the minimum angle of resolution, NA = not applicable, CF = count fingers.

Our results also showed that FLAK provides a faster visual recovery than conventional PK in the early postoperative period, although the backgrounds of the patients was not completely matched. Although there was no significant differences in the primary diseases between the two groups (p = 0.93), the post-FLAK eyes had more keratoconus as the primary diagnosis. This may contribute to better visual results, faster wound healing, and less comorbidity in the FLAK group than those in the conventional PK group. The geometry of the donor-recipient matching is more physiological due to highly precise and reproducible incision, and requires less tight sutures, resulting in faster visual recovery and less corneal and refractive astigmatism. The donor graft size was slightly smaller in the FLAK group than that in the conventional PK group. The difference in the donor graft size may also contribute to faster visual recovery in the FLAK group. Although we did not usually remove the 10-0 nylon sutures during the 6-month follow-up period in the current study, FLAK enables us to remove the sutures in the early postoperative period, which may be another advantage over conventional PK [13], [16], [17].

Moreover, this is also the first study to evaluate the clinical outcomes of FLAK using the VisuMax femtosecond laser system. There have been only a few studies on FLAK using a straight cut, and all surgical procedures were performed using the Femtec femtosecond laser system (Technolas Perfect Vision GmbH, Germany). [3], [4] Holzer et al firstly reported, in a preliminary study of 5 eyes, that all procedures were performed without any complications. [3] However, no detailed clinical outcomes have been reported in their study. Hoffart et al showed that mean corneal and refractive astigmatism was 4.6±2.3 D and 2.9±1.2 D, respectively, and that mean BSCVA was 20/69 at the last postoperative examination. [4] Their findings of FLAK using the Femtec femtosecond laser was comparable with our results of FLAK using the VisuMax femtosecond laser.

Although the larger size of the donor graft may affect the endothelial cell density in favor of the conventional PK group, we found no significant difference in endothelial cell density between the two groups, suggesting that this femtosecond laser for corneal transplantation itself does not significantly affect endothelial cell density in a clinical setting. Our findings were comparable with previous studies on FLAK, [5], [7]–[9], [11]–[13], [16]–[18] except for one study reported by Hoffart et al [4].

There were at least two limitations to this study. Firstly, preoperatively, the items in the patient backgrounds were not completely matched. A prospective randomized controlled study is necessary to confirm the authenticity of the results. Secondly, the sample size is relatively small with a short follow-up. However, the sample size in this study offered >80% statistical power at the 5% level. A further long-term study employing this femtosecond laser technology is required in order to clarify this point.

In summary, this comparative study indicates that FLAK using VisuMax femtosecond laser system induces significantly less corneal and refractive astigmatism than conventional PK, and that it provides significantly faster visual recovery than conventional PK in the early postoperative period, possibly because the geometry of the donor-recipient matching is more physiological and requires less tight sutures. FLAK may have advantages over conventional PK in terms of astigmatism and fast visual recovery. Further long-term studies with a far greater number of subjects are required in order to confirm our preliminary findings.
